# The role of CTCF in the organization of the centromeric 11p15 imprinted domain interactome

**DOI:** 10.1093/nar/gkab475

**Published:** 2021-06-09

**Authors:** Natali S Sobel Naveh, Daniel F Deegan, Jacklyn Huhn, Emily Traxler, Yemin Lan, Rosanna Weksberg, Arupa Ganguly, Nora Engel, Jennifer M Kalish

**Affiliations:** Division of Human Genetics, Children's Hospital of Philadelphia, Philadelphia, PA 19104, USA; Fels Institute for Cancer Research and Molecular Biology, Temple University School of Medicine, Philadelphia, PA 19140, USA; Fels Institute for Cancer Research and Molecular Biology, Temple University School of Medicine, Philadelphia, PA 19140, USA; Division of Human Genetics, Children's Hospital of Philadelphia, Philadelphia, PA 19104, USA; Epigenetics Institute, Department of Cell and Developmental Biology, Perelman School of Medicine, University of Pennsylvania, Philadelphia, PA 19104, USA; Division of Clinical and Metabolic Genetics, Genetics and Genome Biology, Hospital for Sick Children, and Institute of Medical Science, University of Toronto, Toronto, Canada; Department of Genetics, Perelman School of Medicine at the University of Pennsylvania, Philadelphia, PA 19104, USA; Fels Institute for Cancer Research and Molecular Biology, Temple University School of Medicine, Philadelphia, PA 19140, USA; Division of Human Genetics, Children's Hospital of Philadelphia, Philadelphia, PA 19104, USA; Department of Genetics, Perelman School of Medicine at the University of Pennsylvania, Philadelphia, PA 19104, USA; Department of Pediatrics, Perelman School of Medicine at the University of Pennsylvania, Philadelphia, PA 19104, USA

## Abstract

DNA methylation, chromatin-binding proteins, and DNA looping are common components regulating genomic imprinting which leads to parent-specific monoallelic gene expression. Loss of methylation (LOM) at the human imprinting center 2 (IC2) on chromosome 11p15 is the most common cause of the imprinting overgrowth disorder Beckwith-Wiedemann Syndrome (BWS). Here, we report a familial transmission of a 7.6 kB deletion that ablates the core promoter of *KCNQ1*. This structural alteration leads to IC2 LOM and causes recurrent BWS. We find that occupancy of the chromatin organizer CTCF is disrupted proximal to the deletion, which causes chromatin architecture changes both in *cis* and in *trans*. We also profile the chromatin architecture of IC2 in patients with sporadic BWS caused by isolated LOM to identify conserved features of IC2 regulatory disruption. A strong interaction between CTCF sites around *KCNQ1* and *CDKN1C* likely drive their expression on the maternal allele, while a weaker interaction involving the imprinting control region element may impede this connection and mediate gene silencing on the paternal allele. We present an imprinting model in which *KCNQ1* transcription is necessary for appropriate CTCF binding and a novel chromatin conformation to drive allele-specific gene expression.

## INTRODUCTION

Genomic imprinting is the parent-specific monoallelic expression of a subset of mammalian genes. Many imprinted genes play a role in fetal or neonatal development (1,2). In general, paternally-expressed genes promote growth leading to larger offspring, while maternally-expressed genes promote growth restriction and smaller birth weight (2). Marking of the paternal and maternal alleles to differentiate between the two is achieved through epigenetic mechanisms, including DNA methylation and histone post-translational modifications, which are established in germ cells while the genomes are in separate compartments (3). Misregulation of the epigenetic marks and/or expression of imprinted loci often leads to growth or developmental disorders (4).

Beckwith–Wiedemann Syndrome (BWS, OMIM 130650) is one such human imprinting disorder. BWS has recently been revised as the Beckwith–Wiedemann Spectrum (BWSp), which comprises a range of fetal and neonatal overgrowth phenotypes, including macroglossia, omphalocele, organomegaly and embryonal tumors (5–7). The incidence of BWS has been estimated at 1/10 000 live births (8). A small percentage (∼3%) of patients are diagnosed with BWS due to a structural abnormality within the *KCNQ1* gene (5,9,10). Deletions, translocations, and duplications encompassing the imprinting control region (ICR) at the centromeric *KCNQ1OT1:TSS-DMR*, also known as imprinting center 2 (IC2), located on chromosome 11p15 have been reported (9,11–16). More rarely, structural abnormalities outside of the ICR are observed (17–19).

Approximately half of all patients with BWS are molecularly diagnosed with isolated loss of methylation (LOM) at IC2 (5). Within this imprinting cluster, *cyclin-dependent kinase inhibitor 1C (CDKN1C)* and *potassium voltage-gated channel subfamily Q member 1 (KCNQ1)* are maternally-expressed, while the non-coding *KCNQ1* antisense transcript *KCNQ1OT1* is paternally-expressed in the embryo proper (20–24). A maternally-methylated CpG island located at the 5′ end of *KCNQ1OT1* and within intron 10 of the *KCNQ1* gene acts as the differentially methylated region (DMR) for this ICR (22,25–27). Maintenance of this methylation and, subsequently, imprinted gene expression is also dependent on the parent-specific gene expression of both *KCNQ1* and *KCNQ1OT1* (20,28–33).

While aspects of the mechanism that regulates this locus have been investigated, for example the *Kcnq1ot1* transcript recruitment of Dnmt1, Ezh2, Prc2 and G9a (31,34–36), the sequential steps responsible for organizing and establishing the IC2 imprint have yet to be elucidated. As such, interest in investigating the importance of the three-dimensional (3-D) organization at this locus has been growing. Premature termination of *Kcnq1ot1* was found to alter chromatin conformation capture (3C) interactions in the mouse heart (31,37,38) and, more directly, *Kcnq1ot1* silencing abrogates a long-range interaction between the DMR and the *Kcnq1* promoter (36).

One major player in the long-range organization and transcription of imprinted clusters is CCCTC-Binding Factor (CTCF) (39). Within the telomeric 11p15 domain of *H19-ICR*, also known as imprinting center 1 (IC1), CTCF plays a role in methylation maintenance (40), imprinted gene expression (41), and the 3D conformation underlying this domain (42). Although its exact role in IC2 imprinting is as yet unknown, CTCF binding across the region has been previously reported. CTCF binds to *CDKN1C* to modulate cell-specific expression (43–45) and potentially to the DMR itself in a methylation-dependent manner (44–46). Within *KCNQ1* intron 2, there are two additional sites of CTCF occupancy; single nucleotide polymorphisms (SNPs) at these sites reduce CTCF binding affinity and confer risk of IC2 LOM (47) and the region has been suggested as a potential *CDKN1C* enhancer (48). Lopez Abad and colleagues demonstrated this region interacts with *CDKN1C* in human placenta (49). Recently, a study by Rovina *et al.* demonstrated that interaction between the DMR and the *KCNQ1* intron 2 CTCF sites is significantly reduced in BWS patient cell lines (50). Together, these results suggest that CTCF performs an important function in organizing the imprinted locus and may precede initial deposition of DNA methylation or play a role in maintaining DNA methylation.

Here, we report a familial case of BWS transmitted through a deletion at the 5′ end of the *KCNQ1* gene, outside of the ICR. Similar to the cases reported by Beygo *et al.* (19) and Demars *et al.* (17), the structural abnormality a distance away from the DMR leads to LOM at the DMR. We investigate the CTCF occupancy outside of the IC2 DMR and its role in chromatin organization of this imprinted domain.

## MATERIALS AND METHODS

### Patient samples

Samples and clinical information were collected under a previously established Institutional Review Board protocol (IRB 13-010658) at the Children's Hospital of Philadelphia. Consent was obtained from all patients and/or their guardians to collect clinical information and samples. Skin samples were collected from patients and fibroblasts were cultured as previously described (51,52). Briefly, skin samples were split, with one section chemically disrupted using collagenase and the other mechanically disrupted using a scalpel blade to mince. Both explants were seeded into a T25 flask and fed with RPMI skin media (RPMI with fetal bovine serum, penicillin–streptomycin antibiotic, and a final concentration of 2 mM l-glutamine). Flasks were incubated at 37°C for up to one month, with periodic media changes. Successful explant cultures were trypsinized and passaged for sustained growth, then frozen down and stored in liquid nitrogen. Clinical testing for BWS was performed at the University of Pennsylvania Genetic Diagnostic Laboratory as previously described (53).

Fibroblast and placenta/amniocyte samples collected from patients clinically diagnosed with BWS due to isolated LOM at IC2 and not caused by a structural alteration are identified as BWS LOM. Control fibroblast samples were collected from patients who were not diagnosed with BWS and are identified as controls 1–3. Control placenta samples were collected from the birth of BWS LOM patients 3–5 siblings that themselves did not present with BWS features. Placenta tissue was flash frozen for storage at –80°C. Methylation results are reported for amniocytes in place of placenta where such a test was clinically performed and results were available for research use to conserve limited tissue samples.

### Whole genome sequencing (WGS)

Genomic DNA (gDNA) was isolated from control 3 and III-3 fibroblasts using the AllPrep DNA/RNA Micro Kit (QIAGEN) per manufacturer's instructions, then quantified using the Qubit dsDNA HS Assay kit (Invitrogen). Libraries were prepared at the Children's Hospital of Philadelphia Center for Applied Genomics using Enzymatic Fragmentation and Twist Universal Adapter System (Twist Bioscience) with 200 ng of gDNA input as per the manufacturer instructions with the following modifications: 2 PCR cycles were used instead of the recommended 8; 14 min of fragmentation time was applied instead of the recommended 22 min. The TapeStation 4200 (Agilent) was used to quality check the libraries, which were then sequenced on the NovaSeq SP platform (Illumina) across two lanes to achieve 30× coverage. Reads were deduplexed and quality assessed using FastQC (Andrews, S. (2010) FastQC: a quality control tool for high throughput sequence data). Mapping was performed using standard BWA MEM variables (54) to the human hg19 assembly. Reads from both lanes were pooled and visualized in .bam and .bedgraph formats using IGV (55).

### IC2 expression analysis

RNA was extracted from fibroblasts and placenta using the AllPrep DNA/RNA Micro Kit (QIAGEN) per manufacturer's instructions. cDNA was synthesized using the iScript™ cDNA Synthesis kit (Bio-Rad) with a consistent amount of RNA (500 ng for placenta and 70 ng for fibroblasts), assessed by the Qubit RNA HS Assay kit (Invitrogen) for each sample. Primers were manufactured by Integrated DNA Technologies and the primer sequences are listed in Supplementary Table S1. qRT-PCR gene expression quantification was performed using iTAQ SYBR (Bio-Rad) on the Bio-Rad CFX96 Touch Real-Time PCR Detection System with the following condition changes: 58°C annealing/extension. Results of the qPCR were analyzed using the Δ ΔC_T_ method (56).

### Publicly available data usage

CTCF chromatin immunoprecipitation sequencing (ChIP-seq) data available through the ENCODE project (57,58) was viewed using the UCSC genome browser (59) from the following UCSC accession codes for human hg19 build: hESC1 wgEncodeEH000085 for CTCF, wgEncodeEH000106 for H3K4me1, wgEncodeEH000086 for H3K4me3, wgEncodeEH000997 for H3K27ac, wgEncodeEH000074 for H3K27me3; and for mouse mm9 build: wgEncodeEM001954 for CH12 CTCF. Mouse allele-specific CTCF binding ChIP-seq files (GSM862560 and GSM862561) (60) were accessed through NCBI GEO, downloaded through NCBI SRA run selector, and BAM files were viewed directly with IGV (55). BAM files were also converted to bedgraph for viewing on UCSC Genome Browser through the use of Bedtools2 (61). Hi-C data available through the ENCODE project was accessed through the 3D Genome Browser (62) from Lieberman-raw for the GM12878 human hg19 build and CH12 mouse mm9 build (63).

### Chromatin immunoprecipitation (ChIP)

ChIP was performed on chromatin isolated from patient-derived fibroblasts using the MAGnify Chromatin Immunoprecipitation System (Invitrogen) as per manufacturer recommendation. Chromatin was sonicated using the Covaris ME220 with the following parameters: PIP 75, DF 5%, CPB 200, 6°C setpoint for 16 min total, then precipitated using anti-CTCF (ab70303; Abcam) and rabbit IgG. To quantify CTCF occupancy, qPCR was performed with primers listed in Supplementary Table S1 using iTAQ SYBR (Bio-Rad) on the Bio-Rad CFX96 Touch Real-Time PCR Detection System with the following conditions: 58°C annealing/extension. Results of the qPCR were analyzed using the ΔΔC_T_ method (56).

### Sanger sequencing

Sanger sequencing was used to perform allele-specificity experiments. PCR products were amplified from one of three templates as specified: (i) gDNA isolated from fibroblasts using the AllPrep DNA/RNA Micro Kit (QIAGEN) per manufacturer's instructions, (ii) cDNA created as described in the expression analysis and (iii) ChIP DNA generated in the CTCF immunoprecipitation. Amplicons were run on an agarose gel and subsequently gel extracted using the Gel Extraction kit (QIAGEN) per manufacturer's instructions. Sequencing was carried out on the Applied Biosystems 3730xl DNA Analyzer platform (Thermo Fisher). Primers were manufactured by Integrated DNA Technologies and the primer sequences are listed in Supplementary Table S1.

### Capture C

Capture-C was performed according to the protocol from Davies *et al.* (64) using the restriction enzyme DpnII (NEB) with the following modifications. After each step, the concentration and size distribution of the samples was determined using the Bioanalyzer 2100 (Agilent) unless otherwise noted. After cross-linking, samples were sonicated using the Covaris S220 with the following parameters: PIP 5, DF 10%, CPB 200, 7°C setpoint for two 60 s cycles. NEBNext Multiplex Oligos for Illumina (NEB) were used in adaptor ligation with the following modifications: 0.1× TE buffer was used for all elution steps, LoBind PCR plates (Eppendorf) were used for dA-Tailing of End Repaired DNA and subsequent steps, adaptor ligated DNA was size selected for 200 bp fragments using AMPure XP beads (Beckman Coulter), and a total of 6 cycles were employed for PCR enrichment of the adaptor ligated DNA. Adaptor ligated DNA samples were mixed together in equal amounts by mass to obtain two separate pools of 1.5–2ug: one pool containing three samples and the other containing four.

Probes, whose sequences are listed in Supplementary Table S1, were obtained as 4 nmol biotinylated oligonucleotide IDT ultramers and were resuspended to a final concentration of 2.9 uM. Equimolar amounts of these probes were then pooled to use for the oligonucleotide capture. The capture was performed with the SeqCap EZ Library Kit (Roche) as per manufacturer's instructions, with the following modifications: Capture Beads provided in the SeqCap Pure Capture Bead Kit and KAPA HiFi HotStart ReadyMix from the SeqCap EZ Accessory Kit V2 were used. Two rounds of capture were performed with only 75% (up to 2 ug) of captured material used in the second capture and the hybridization was performed for 24 h instead of 64–72 h as in the first capture. Before sequencing, the concentration, quality, and size of the final captured material was determined by Qubit dsDNA HS Assay (Invitrogen) and the Bioanalyzer 2100 (Agilent). Samples were sequenced on the HiSeq platform (Illumina) with a 2 × 150 bp run format.

Paired-end reads were trimmed with Trim Galore (version 0.4.4_dev) (http://www.bioinformatics.babraham.ac.uk/projects/trim_galore/) using default parameters. FLASH (v1.2.11) (65) was then used to merge paired-end reads from the same fragment, allowing the reads to overlap to a maximum of 150 bp (‘-M 150′). Fragments were digested with DpnII using the Python script implemented by the CCanalyser3 pipeline (64). The digested fragments were mapped to hg19 reference genome using Bowtie (version 1.2.2) (66), during which reads with more than two alignments were suppressed and only the best alignment was reported (‘-m 2 –best’). The paired-end relationship between the reads were re-established, PCR duplicates were removed, and read enrichment was quantified by the CCanalyser3 pipeline. Contact sites in each sample were defined by fourSig (67). Signal for each captured region was quantified on each bigWig file generated from the pipeline using bwtool (v1.0) (68). *cis*- and *trans*-interactions were filtered using Bedtools2 (61) to capture signals common between biological replicates within each group. Figures depicting these cis- and trans-interactions were generated using the R packages Sushi (69) and Circlize (70), respectively.

## RESULTS

### Characterization of a case of familial BWS

**Figure 1.**
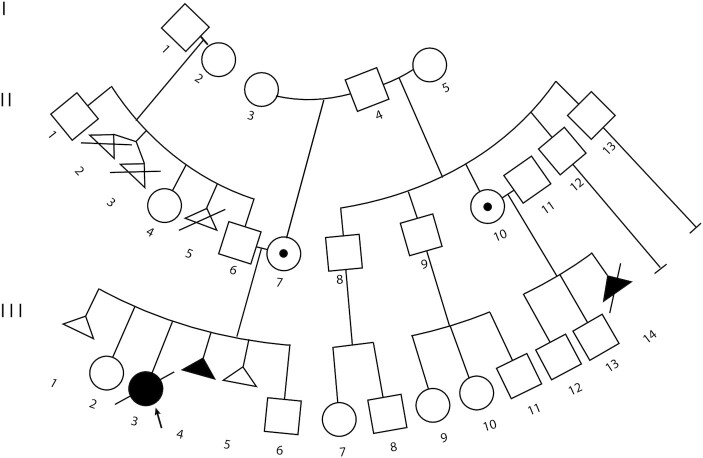
Familial inheritance of a *KCNQ1* 5′ deletion. Three generation pedigree of the familial transmission of BWS. Arrow indicates proband (III-3). Affected family members in black shapes (individuals III-3, III-4 and III-14); dots in the shape indicate unaffected deletion carriers (individuals II-7 and II-10). These carriers and affected family members are related through a common maternal father and grandfather, respectively (individual I-4).

The index patient or proband (III-3) was a female born at 26 6/7 weeks to a 29-year old (II-7) by natural conception (Figure 1). There was a maternal history of a first trimester spontaneous miscarriage (Figure 1). An omphalocele and enlarged adrenal glands were detected in the second trimester leading to BWS testing on amniocytes that showed IC2 LOM (Table 1). Birth weight was 1.16 kg (75th percentile) and postnatal exam demonstrated macroglossia, ear crease, omphalocele, but no lateralized overgrowth, features all consistent with the diagnosis of BWS. Placental pathology showed mesenchymal dysplasia and initial abdominal ultrasound showed cortical renal cysts. The patient passed away at 2 days due to complications of prematurity. Additionally, the maternal half-aunt of the proband, through a common grandfather II-10, was found to carry a fetus (III-14) with a prenatal diagnosis of placentomegaly, nephromegaly, and micrognathia, without polyhydramnios on a 22-week anatomy ultrasound (Figure 1). Given the likelihood of severe BWS based on the index case, the family opted for termination.

**Table 1.** Methylation levels at IC1 and IC2 in controls, BWS LOM patients and IC2 deletion family members. Normal range for IC1 is 45–55% and for IC2 is 46–54% as per reported error rate

	Sample type	IC1%	IC2%	Deletion carrier
Control1	BLOOD	49.49	51.43	N/A
Control2	SKIN	49.85	50.27	N/A
Control3	SKIN	50.55	50.72	N/A
BWS LOM1	BLOOD	50.91	2.43	N/A
BWS LOM2	BLOOD	48.16	0.05	N/A
BWS LOM3	BLOOD	48.36	0.05	N/A
BWS LOM3	AMNIOCYTES	53.13	2.78	N/A
BWS LOM4	PLACENTA	50.00	0.02	N/A
BWS LOM5	AMNIOCYTES	49.03	22.54	N/A
Familial deletion I-4	BLOOD	51.04	50.87	Likely germline mosaic
Familial deletion II-6	BLOOD	52.49	51.01	No
Familial deletion II-7	BLOOD	49.55	50.87	Yes
Familial deletion II-10	BLOOD	51.04	50.54	Yes
Familial deletion II-11	BLOOD	49.39	50.44	No
Familial deletion III-2	BLOOD	43.73	50.36	No
Familial deletion III-3	BLOOD	51.01	0.01	Yes
Familial deletion III-3	AMNIOCYTES	49.12	0.04	Yes
Familial deletion III-14	SKIN	51.52	0.01	Yes
Familial deletion III-14	PLACENTA	51.86	0.01	Yes

**Figure 2.**
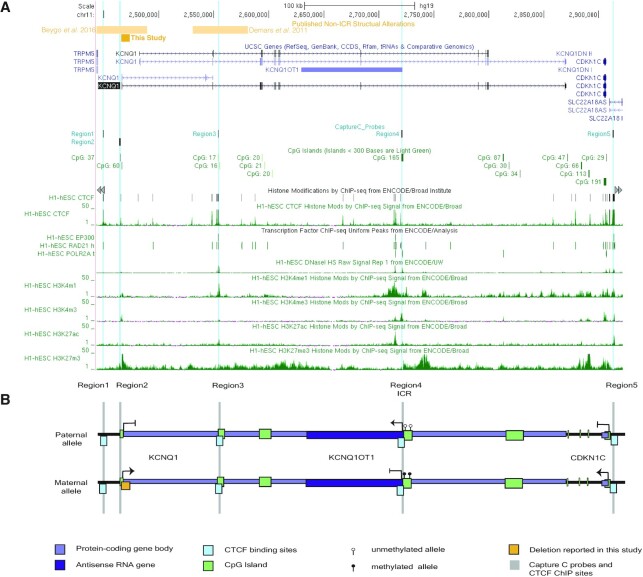
Diagram of *KCNQ1, KCNQ1OT1*, and *CDKN1C* within the IC2 domain. (**A**) UCSC genome browser view of hg19 chr11:2 443 460–2 921 770 including ENCODE tracks of histone modifications and DNase hypersensitivity (HS) sites observed in H1-hESC samples. (**B**) A stylized diagram of the IC2 domain demonstrating the parent-of-origin gene expression in scale with the UCSC view. In both panels. The documented BWS-causing *KCNQ1* 5′ deletion is indicated by the gold box. Protein-coding genes (*KCNQ1* and *CDKN1C)* antisense transcript (*KCNQ1OT1)* are indicated in purple. CpG islands within the region are displayed in green, while CTCF binding sites are highlighted in aqua. Capture C anchors and ChIP-qPCR primer sites are indicated by Region1-5 labels. Scale bar indicates 100 kb.

Methylation testing on chromosome 11p15 was performed on family members (Table 1). All samples were found to have normal methylation, ∼50%, at the telomeric *H19-ICR* (IC1) (Table 1). While most family members also presented with normal methylation at IC2, again ∼50%, samples from both the proband III-3 and individual III-14 presented with complete depletion of methylation at this centromeric domain (Table 1). However, as LOM is usually a mosaic somatic event and not likely to present with familiar transmission, we used Agilent custom microarrays as previously described (53) to verify the previous MS-MLPA copy number results. Using this technique, a small 6.8 kb deletion was identified (chr11: 2 466 678–2 473 512 [grch37/hg19]) (Supplementary Figure S1A). To more finely map the breakpoints of this deletion, we performed Whole Genome Sequencing (WGS) on fibroblasts derived from patient III-3 as well as fibroblasts derived from a non-BWS patient, control3 (Table 1). Read counts were decreased by approximately 50% along the chr11:2 466 050–2 473 630 interval in the III-3 sample (Supplementary Figure S1B) that are unlikely to be a sequencing artifact based on the mapped read count in the control3 profile (data not shown). This result indicates that the full deletion stretches nearly 7.6 kb from approximately 200 bp upstream of the *KCNQ1* transcription start site (TSS) and into its first intron, thereby spanning the *KCNQ1* core promoter (Figure 2).

### Transcription of genes within the centromeric 11p15 imprinted domain

**Figure 3.**
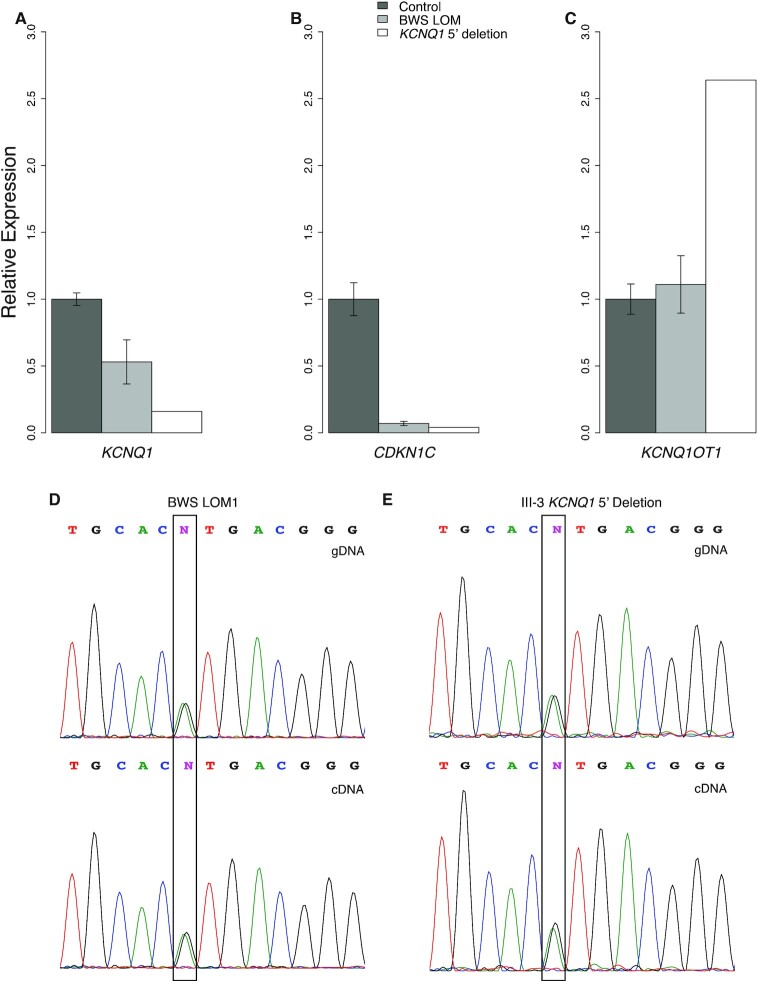
Expression of *KCNQ1, CDKN1C*, and *KCNQ1OT1* imprinted genes within IC2. Relative expression of IC2 imprinted genes was assessed by qRT-PCR and normalized to *ACTB* and *RPLP0* levels. For LOM, *N* = 3; for control, *N* = 3. Error bars represent CI. (**A**) Expression of maternally-expressed *KCNQ1* in placenta is reduced in BWS LOM cells and is further disrupted in III-3 cells carrying the maternal allele deletion relative to control samples from BWS LOM siblings. (**B**) Expression of maternally-expressed *CDKN1C* in fibroblasts is similarly aberrantly repressed in both BWS LOM and III-3 cells relative to unrelated non-BWS controls. (**C**) Expression of paternally-expressed *KCNQ1OT1* in fibroblasts is increased in III-3 cells carrying the *KCNQ1* 5′ deletion relative to unrelated non-BWS controls. (**D**) Sanger sequencing trace of a *KCNQ1OT1* SNP, rs231362 designated by the black box, in genomic (top) and complementary (bottom) DNA isolated from BWS LOM1 fibroblasts demonstrates biallelic expression. (**E**) Sanger sequencing trace of the boxed rs231362 SNP in genomic (top) and complementary (bottom) DNA isolated from III-3 *KCNQ1* 5′ deletion fibroblasts demonstrates biallelic expression.

As the observed familial deletion appears to ablate the core promoter of *KCNQ1* (Figure 2), we wanted to determine the impact of this structural abnormality on its transcription level. Transcription levels of the maternally-expressed *KCNQ1* are low in fibroblast samples, which are used in subsequent experiments; to effectively assess its expression by quantitative reverse transcription PCR (qRT-PCR), we used placenta, a tissue with moderate transcript abundance (71) (Figure 3A). For this assay, BWS LOM patient 3, 4 and 5 placentae are represented in the BWS LOM group (Table 1, Figure 3A). Controls used in this assay were collected from siblings of BWS LOM 3, 4 and 5 who did not present with BWS (Figure 3A). Approximately 50% decreased expression of *KCNQ1* was observed in BWS LOM samples relative to the non-syndromic sibling samples. In the III-3 *KCNQ1* 5′ deletion sample, ∼85% reduced *KCNQ1* expression was observed (Figure 3A). These results indicate that maternal *KCNQ1* transcription is disrupted further in individual III-3 carrying the maternal deletion as compared to the isolated LOM patients. Some of the moderate *KCNQ1* transcription detected in III-3 placenta may be due to maternal contamination in tissue processing, but a study in mouse demonstrated a small amount of transcription is detected from the paternal allele in late-term placenta (34). In either event, *KCNQ1* expression is effectively maternally-silenced by the deletion at the 5′ end of this gene.

Additionally, maternal methylation at the IC2 DMR, located at the 5′ end of *KCNQ1OT1*, was affected by the *KCNQ1* 5′ deletion (Table 1). As this methylation is important in the imprinted expression of *KCNQ1, KCNQ1OT1*, and *CDKN1C*, we wanted to determine the expression status of *KCNQ1OT1* and *CDKN1C* as well (Figure 2B). We determined imprinting maintenance of *CDKN1C* and *KCNQ1OT1* in fibroblasts for BWS LOM patients 1, 2, 3 and III-3 (Table 1), as this cell type was used in subsequent experiments in this study (Figure 3B-C). For these qRT-PCR experiments, controls were derived from unrelated non-BWS patients (Table 1, Figure 3B and C). Transcription of the maternally-expressed *CDKN1C* was ablated, with 7% and 4% expression in BWS LOM and III-3 fibroblasts respectively, compared to that of the control group (Figure 3B). Conversely, transcription of the paternally-expressed *KCNQ1-*antisense *KCNQ1OT1*, which initiates from the ICR, is more than doubled in fibroblasts from patient III-3 although expressed at similar levels between BWS LOM and control groups (Figure 3C). Due to loss of maternal methylation in both BWS LOM and III-3 *KCNQ1* 5′ deletion samples, we wanted to determine whether expression of *KCNQ1OT1* was biallelic, irrespective of transcript abundance (Figure 2B). We performed Sanger sequencing to assay for allele-specific polymorphisms in *KCNQ1OT1* transcripts (Figure 3D and E). In the sample isolated from BWS LOM1 (Table 1), a single nucleotide polymorphism (SNP), designated rs231362, was captured at the genomic DNA (gDNA) level (Figure 3D). The SNP was also observed in *KCNQ1OT1* complementary DNA (cDNA) (Figure 3D), indicating biallelic expression of this antisense RNA. The rs231362 polymorphism was also detected in III-3 fibroblasts at the gDNA and cDNA levels (Figure 3E), suggesting loss of maternal *KCNQ1* expression causes reactivation of the maternal *KCNQ1OT1* antisense transcript as a result of the deletion.

### CTCF occupancy within the centromeric 11p15 imprinted domain

We wanted to understand the mechanism of this allele-specific expression disruption and hypothesized that CTCF binding across the region may play a role. CTCF has been shown to bind at *CDKN1C* (43,47), but observations as to whether it binds at the ICR have been conflicting (30,36,46,60,72). While the importance and consistency of occupancy at the DMR is questionable, other CTCF binding sites have been observed across the imprinted domain through chromatin immunoprecipitation sequencing (ChIP-seq) experiments (Figure 2) and in targeted ChIP studies (47,49).

As the orthologs of *KCNQ1, KCNQ1OT1* and *CDKN1C* are also subject to genomic imprinting in the mouse (21,25,73), we wanted to determine the landscape of CTCF binding across the orthologous domain. Again, murine occupancy of CTCF on the ICR has previously been investigated and results include the finding that CTCF binds biallelically (72), to only the paternal allele (46), or not at all (60). Outside of the ICR, murine ChIP-seq profiles indicate CTCF binds upstream of *Kcnq1*, within the *Kcnq1* intron2, and upstream of *Cdkn1c* (Supplementary Figure S2A). To ascertain whether this binding is allele-specific in the mouse, we used a publicly available CTCF ChIP-seq dataset (60). In the previously published work, Prickett and colleagues determined that CTCF does not bind appreciably to the ICR of IC2, but our visualization of their data indicates CTCF binds to sites upstream of *Kcnq1*, strongly within the *Kcnq1* gene body, and upstream of *Cdkn1c* (Supplementary Figure S2A). To determine whether this binding was monoallelic or biallelic, we visualized SNPs present within these binding sites (Supplementary Figure S2B). Variants were observed in approximately equal proportion by sequencing, indicating equal interaction of CTCF with both alleles (Supplementary Figure S2B).

**Figure 4.**
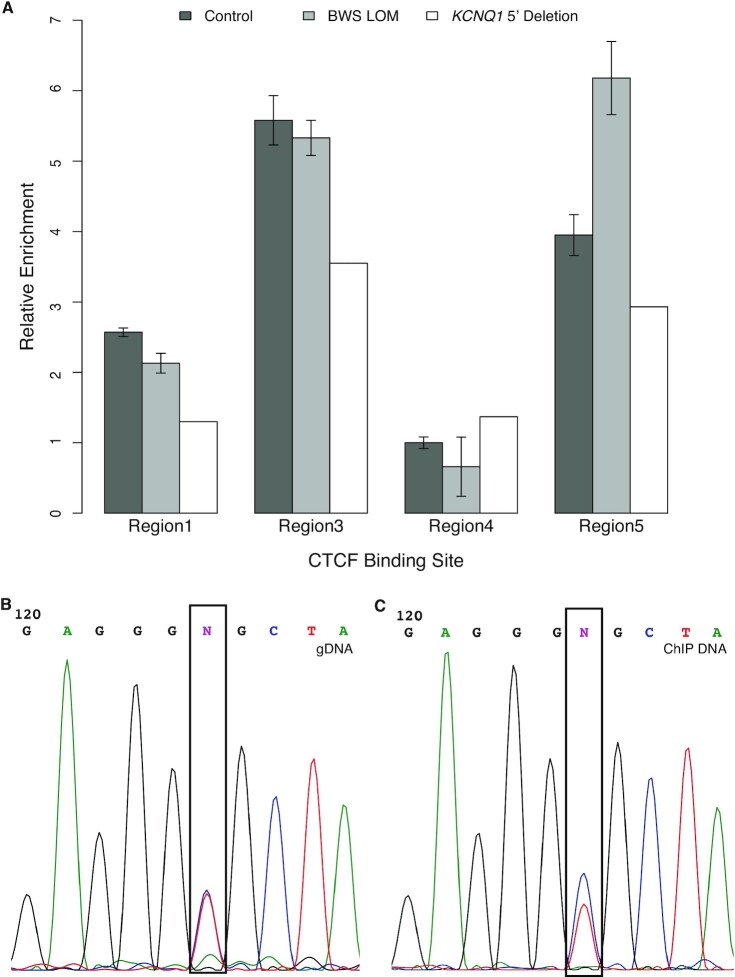
CTCF binding across the IC2 deletion. ChIP-qPCR results of CTCF occupancy relative to the non-imprinted *ANXA9* locus. For BWS LOM, N = 3; for unrelated non-BWS controls, N = 3. Error bars represent CI. (**A**) CTCF binding at Regions 1, 3, 4 and 5 relative to the control level of binding at the Region4 ICR. Regions 1, 3 and 5 have higher CTCF occupancy than that at Region4. Relative to controls, III-3 fibroblasts carrying the *KCNQ1* 5′ deletion demonstrate reduced CTCF binding Regions 1 and 3. (**B**) Sanger sequencing trace of the rs67439072 SNP designated by the black box in genomic DNA isolated from III-3 *KCNQ1* 5′ deletion fibroblasts located less than 100 bp from the Region1 CTCF site. (**C**) Sanger sequencing trace of the same boxed SNP in ChIP DNA isolated from III-3 *KCNQ1* 5′ deletion fibroblasts.

To determine whether CTCF binds to similar sites across the human IC2 domain and whether this binding was disrupted by the *KCNQ1* 5′ deletion, termed Region2 (Figure 2), we performed chromatin immunoprecipitation followed by quantitative PCR (ChIP-qPCR). Comparable to the sites observed in mouse, we targeted CTCF sites near the deletion upstream of *KCNQ1* termed Region1 as well as within *KCNQ1* intron 2 termed Region3 (Figure 2). In addition, we assessed CTCF binding at the ICR termed Region4, and a site ∼500 kb from the deletion at the 5′ end of *CDKN1C* termed Region5 (Figure 2). While CTCF was detected at the Region4 ICR, the level of occupancy at this site was relatively low across the control1-3, BWS LOM1-3 and III-3 fibroblast samples (Figure 4A). Additionally, there were no appreciable differences in CTCF binding level between groups at this site (Figure 4A). CTCF binding sites at Regions 1, 3 and 5 all demonstrated greater relative enrichment, with the most CTCF detected at the Region3 binding motif in controls (Figure 4A). Similar levels of occupancy were detected at Regions 1 and 3 between the control and BWS LOM groups (Figure 4A). While there was some increased CTCF binding to Region5 in BWS LOM samples relative to control, this did not reach a 2-fold increase (Figure 4A). Decreased CTCF binding was observed in the III-3 deletion fibroblasts with 50–60% of control-level enrichment at both Regions 1 and 3 (Figure 4A).

As the III-3 *KCNQ1* 5′ deletion is only present on the maternal allele, we wanted to determine whether CTCF occupancy was also monoallelic in this fibroblast sample. We performed Sanger sequencing on amplicons generated from gDNA and from the CTCF ChIP DNA to detect allelic SNPs (Figure 4B and C). In this sample, a SNP was detected within the Region1 CTCF site, designated rs67439072, at the genomic level (Figure 4B). Visualization of the same sequence in the ChIP DNA indicated the SNP was still present, suggesting the capture of both alleles by the CTCF immunoprecipitation (Figure 4C). There was a bias towards capture of the C allele, which may indicate preferential loss of CTCF on one allele, but certainly not complete loss of CTCF in an allele-specific manner (Figure 4C). Overall, these results indicate that high biallelic CTCF binding occurs at sites across the domain and the structural abnormality observed in the III-3 samples may somehow influence this binding.

### Cis and trans interactions with the centromeric 11p15 imprinted domain

**Figure 5.**
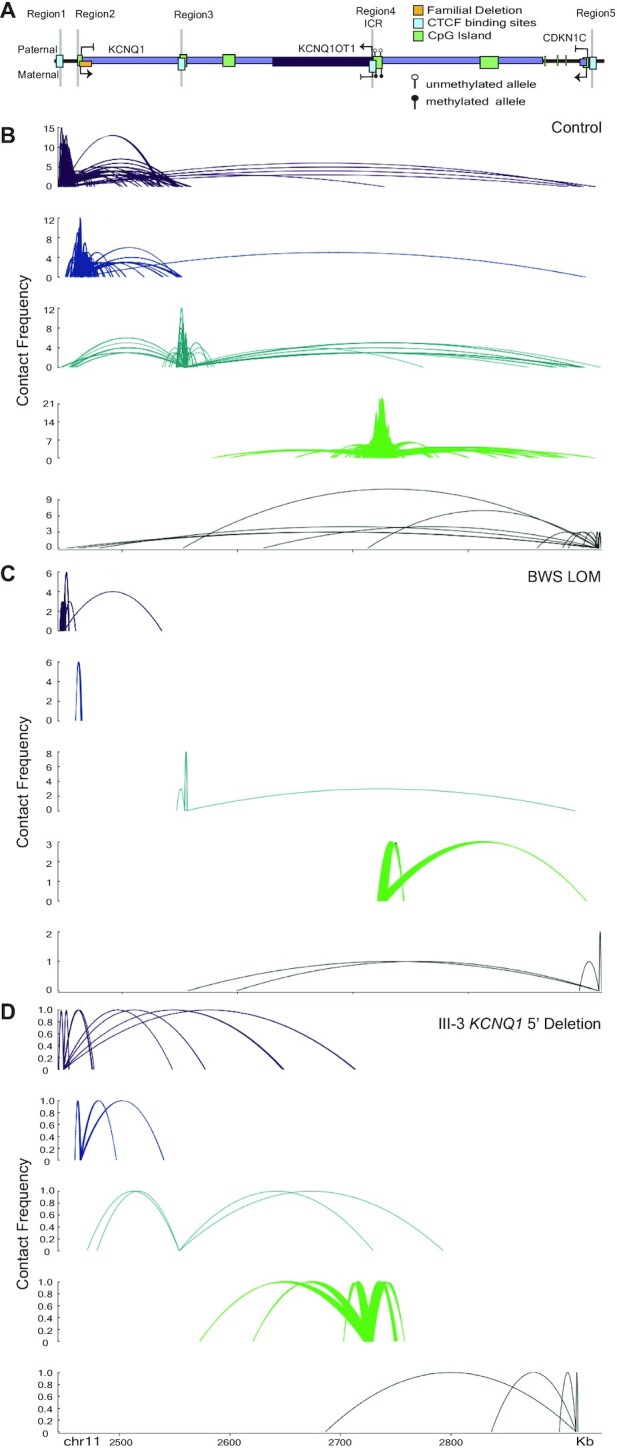
Interaction profiles between 5′ *KCNQ1*, IC2, and 5′ *CDKN1C* change after BWS LOM and *KCNQ1* 5′ deletion. For LOM, *N* = 3; for control, *N* = 3. Splines represent interactions commonly observed between biological replicates. Contact frequency indicates the number of times an interaction was observed. (**A**) Simplified locus map indicating the probes for interaction capture. (**B**) *cis* interactions across the IC2 domain in control fibroblasts (*N* = 3) demonstrate interactions between Region1, Region3 and Region5. (**C**) *cis* interactions across the IC2 domain in BWS LOM fibroblasts are reduced, notably connectivity between Region1 and Region5 is absent. (**D**) *cis* interactions across the IC2 domain in III-3 *KCNQ1* 5′ deletion fibroblasts are further reduced in strength compared to BWS LOM, again lacking connectivity between Region1 and Region5.

It has been well-established that CTCF can direct DNA looping and chromatin organization (39). More specifically, CTCF has been observed or proposed to play a role in small-scale interactions across IC2 (36,37,43,49). We performed Capture-C to better understand interactions stemming from the CTCF binding sites, the *KCNQ1* 5′ deletion itself, or the ICR (Figure 5A). First, interactions in the control 1–3 fibroblasts were observed between Region1, Region3 and Region5, with limited connectivity to Region4 (Figure 5B). Interactions between all of these regions are almost completely abrogated in the BWS LOM 1–3 fibroblast samples (Figure 5C). Lastly, in the III-3 deletion fibroblasts, while a few connections between Region4 and Regions1/3/5 remain, the long-range interactions from Region1/3 to Region5 are no longer captured (Figure 5D). Interestingly, these results suggest that the connectivity between the 5′ end of *KCNQ1* and the 5′ end of *CDKN1C*, and to a lesser extent to the ICR, is important to the regulation of imprinting within this domain.

Recently, Rovina *et al.* (50) reported an interaction between IC1 and IC2 in a limited number of patient lymphoblastoid cell lines. To determine whether we observed such an interaction in our patient fibroblast lines, we expanded the window of interactions. In the control fibroblast lines, Region1 was the only anchor to form long-range interactions that extended to the 5′ region of *IGF2*; there were no interactions observed between the Region4 IC2 anchor and IC1 (Supplementary Figure S3B). Trends in the BWS LOM and III-3 deletion samples were similar to those observed within the IC2 domain (Supplementary Figure S3C and D). The BWS LOM fibroblast group lost many connections with all anchors and specifically between Region1 and IC1 (Supplementary Figure S3C), while the III-3 IC2 deletion fibroblasts maintained some connection between Region1/3 and the 5′ end of *IGF2* (Supplementary Figure S3D). Neither of these samples displayed any interactions between Region4 IC2 and IC1 (Supplementary Figure S3C and D). Although we did not confirm the reciprocal interaction using a probe at IC1 to interrogate IC2, these results were supported by Hi-C data that demonstrate a contact depletion indicated by white coloration in a stripe initiating from the middle of the IC1 domain (Supplementary Figure S2A). Further, we wanted to determine whether these aspects of domain organization were conserved in the mouse, as with the CTCF binding. The contact depletion separating the imprinted domains was even more pronounced in the mm9 data (Supplementary Figure S4).

**Figure 6.**
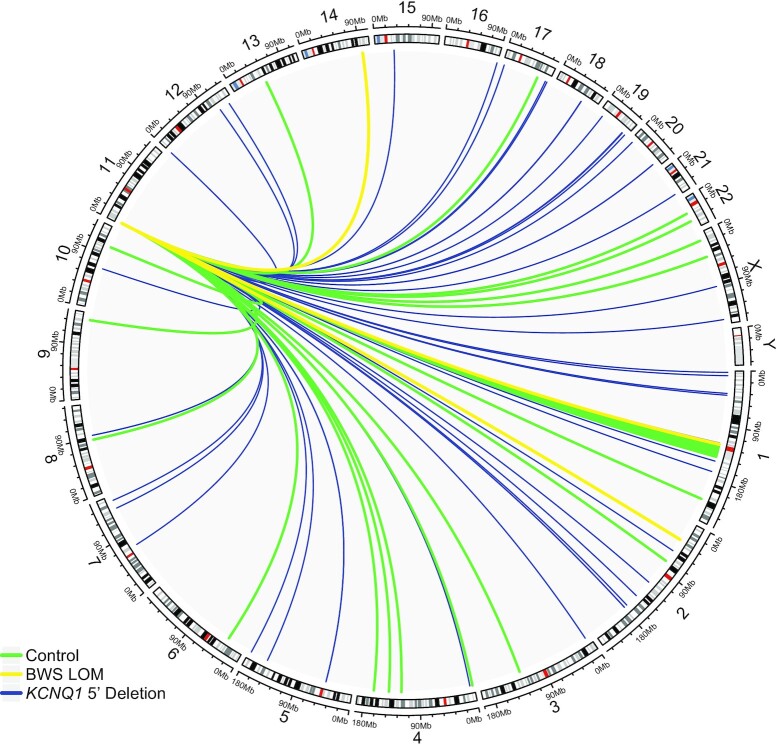
*Trans* interactions of IC2 ICR across the genome. *Trans* interactome of the ICR Region4 probe are displayed. Interactions across chromosome 11 are not shown. Common features of control fibroblasts (*N* = 3) profile are shown in green to establish an average interactome of the ICR when imprinting is maintained. Common features of BWS LOM fibroblasts (*N* = 3) are shown in yellow and indicate that, when imprinting is disrupted, fewer trans interactions are made. The profile of III-3 fibroblasts carrying the *KCNQ1* 5′ deletion on the maternal allele is shown in blue and demonstrates a greater number as well as novel interactions across the genome when imprinting is disrupted and the structure of the imprinted domain is altered.

While we did not observe interactions between IC1 and IC2, we thought there may be *trans* interactions, or associations between other chromosomes and this imprinted domain important to the imprinted expression regulation of IC2 and the etiology of BWS. As such, we first considered interactions between the ICR at Region4 with other chromosomes (Figure 6). Control interactions common among the biological replicates were limited and, as expected, the number of these interactions was severely reduced in the BWS LOM group (Figure 6, Supplementary Table S2). Surprisingly, the number of interactions with this anchor in III-3 *KCNQ1* 5′ deletion fibroblasts was increased both overall as well as the number of chromosomes with which interactions were observed (Figure 6, Supplementary Table S2). This pattern of interaction decreases among the BWS LOM group and increases in the III-3 deletion sample consistently across Region2, Region3, and Region5 probes (Supplementary Figure S5B–D, Supplementary Table S2). Of all the anchors, Region1 had the greatest number of overall interactions and the control and BWS LOM groups presented with a similar quantity of interactions, indicating that a number of these interactions are unlikely to be caused by or to influence imprinting (Supplementary Figure S5A). Together, these results across all anchors point to the importance of previously unexplored *trans* interactions and whole genome organization in regulating genomic imprinting at IC2.

## DISCUSSION

DNA binding proteins, histone post-translational modifications, and chromatin conformation have been shown to play a role in cell-specific and developmental genome regulation (74). Loci subject to parent-of-origin gene expression are also regulated by these epigenetic factors, wherein the maternal and paternal alleles demonstrate distinct intra- and interdomain profiles (75). Here, we investigate aspects of the human centromeric 11p15 imprinted domain epigenetic landscape. Alterations to the structure or ICR-DMR methylation of this domain have been associated with BWSp, a human overgrowth spectrum most commonly caused by the resultant loss of maternal expression of *CDKN1C*.

Part of this profiling includes our presentation of the first *trans*-interactome of IC2 in control and BWS patient fibroblast samples (Figure 6, Supplementary Figure S5). As the control profile is comprised of distinct maternal and paternal allele contacts, we were unsurprised to observe fewer interactions in the BWS LOM group which lost allele-specific regulation (Figure 6, Supplementary Figure S5). Additionally, as the III-3 interactome was not filtered against another biological sample, some of the increased number of interactions in this profile are likely background contacts (Figure 6, Supplementary Figure S5). Although many 11p15 contacts with other chromosomes are likely due to nuclear organization and association of transcriptionally active or inactive domains (76), some may represent functional elements that influence the parent-specific gene expression within IC2. None of the control *trans*-Region5 *CDKN1C* interactions lost in BWS samples displayed the combinatorial histone and DNA binding factor landscape to suggest a potential *CDKN1C* enhancer function (Supplementary Table S2) (57–59). However, one *trans* interaction we noted was that between Region1/Region4 and human chromosome 2p22.3 within intron4 of *LINC00486* (Figure 6, Supplementary Figure S4A, Supplementary Table S2). This contact was observed in the control samples, but not in the BWS samples (Figure 6, Supplementary Figure S4A, Supplementary Table S2). Furthermore, while this long, intergenic, non-coding RNA has not been well-characterized, the interaction contact point overlapped with the signal for histone modifications including H3K4me1 and H3K27ac, as well as binding of CTCF, bHLH transcription factor cMYC, and RNA polymerase II (57–59). In combination, these factors may indicate an enhancer element (77), although whether it acts as such in *trans* on IC2 was not within the scope of this study and requires further exploration. Additionally, while an imprinting gene network has been previously proposed to describe the co-regulation of loci subject to parent-specific gene expression (78,79), we did not observe any connections between any of the IC2 domain anchors and other imprinted loci (Supplementary Table S2).

We report a case of familial BWS caused by the maternal transmission of a 7.6 kB deletion at the 5′ end of *KCNQ1* that causes *KCNQ1* silencing (Figure 3A) and LOM downstream at the ICR (Table 1). Along with the structural abnormalities reported by Beygo *et al.* (19) and Demars *et al.* (17), this deletion is the third observed alteration outside of the ICR-DMR that impacts its methylation status. To determine if long-range intradomain interactions mediated by the chromatin organizer CTCF act within IC2, we profiled CTCF binding and chromatin conformation in control, BWS LOM, and *KCNQ1* 5′ deletion samples (Figure 4–5). To identify common features of IC2 imprint disruption, we compared the III-3 *KCNQ1* 5′ deletion profile to that of the BWS LOM features. While previous studies investigating the CTCF binding profile across the domain have focused on the occupancy at the Region4 ICR (30,36,46,60,72), we find that of the four annotated CTCF binding sites we considered, the control occupancy at the ICR was lowest (Figure 4A). The highest control CTCF binding was observed within Region3 (Figure 4A).

Although our *KCNQ1* 5′ deletion did not encompass any CTCF sites, the two most proximal binding sites, Region1 and Region3, demonstrated reduced CTCF occupancy in this fibroblast sample (Figure 4A). It is possible that *KCNQ1* transcription is required to open Region3 within its intron 2 to allow for CTCF binding to maintain maternal *CDKN1C* expression. The CTCF motif and occupancy at Region3 have been previously investigated in BWS and human imprinting mechanism studies. Risk for IC2 LOM has been correlated to SNP presence within the Region3 CTCF binding sequence (47). Based on epigenetic histone marks, this site was also identified as a potential *CDKN1C* enhancer (Figure 2) (48). The familial duplication reported by Demars *et al.* (17) encompassed this CTCF site and caused IC2 LOM, which resulted in decreased *CDKN1C* expression. Furthermore, an interaction between this Region3 and *CDKN1C* was observed in placenta by Lopez-Abad *et al.* (49). We confirm this physical connection in control fibroblast samples and further conclude that the interaction includes the CTCF site at Region1 nearly 450 kB from *CDKN1C* (Figure 5A-B). It is possible that the presence of a SNP within the binding site impacted CTCF affinity for Region1 in the III-3 sample, although this does not completely explain the capture of both alleles in the ChIP DNA Sanger sequencing (Figure 4C). The interaction between Region1 and Region 3 may also reinforce CTCF binding at both sites; the decrease in CTCF binding at Region3 may influence occupancy at Region1 thereby causing its decrease in III-3 *KCNQ1* 5′ deletion cells (Figure 4A). These long-range connections are depleted in both isolated LOM and *KCNQ1* 5′ deletion samples (Figure 5C-D), implying their importance in the parent-of-origin transcription across the domain.

**Figure 7.**
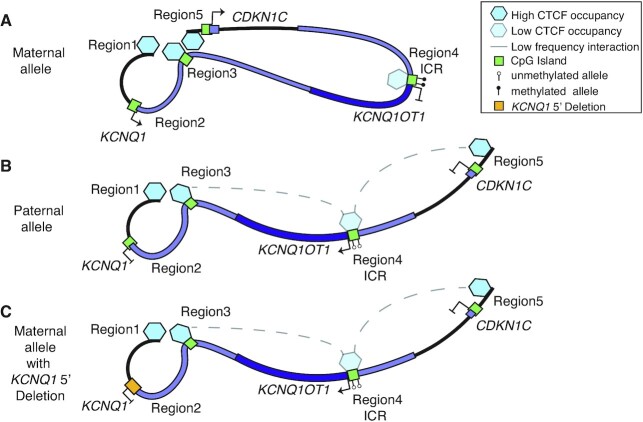
Proposed model of intra-domain looping in IC2 maintenance of imprinting. (**A**) On the maternal allele, interactions connect the 5′ end of *KCNQ1, KCNQ1* intron2 and the 5′ end of *CDKN1C*, which promotes expression of both *KCNQ1* and *CDKN1C*. This looping excludes the ICR. (**B**) On the paternal allele, low frequency contacts between the ICR and the 5′ end of *KCNQ1* and *KCNQ1* intron2 or the 5′ end of *CDKN1C*, but not all three. The result is expression of *KCNQ1OT1*. (**C**) On the maternal allele carrying the *KCNQ1* 5′ deletion, this interaction does not organize correctly. This may be caused by transcription disruption of *KCNQ1*, but leads to an interaction profile resembling the paternal allele and loss of *CDKN1C* expression.

We also observed an interaction between Regions 3/4 and 4/5 that occurred at approximately half the frequency of the control Region1/3/5 connection (Figure 5). This conformation became apparent in the *KCNQ1* 5′ deletion and BWS LOM profiles, respectively, which suggests these interactions were not abrogated by the BWS-associated maternal allele dysregulation. As such, we propose an IC2 looping model wherein CTCF cooperatively mediates the maternal high-strength Regions1/3/5 interaction to bring together the *KCNQ1* and *CDKN1C* TSSs and drive their allele-specific expression (Figure 7). On the paternal allele, the CTCF site at Region4 loops with Region3 or Region5 at a low-frequency, which we propose prevents the formation of the Region1/3/5 loop (Figure 7). Importantly, the results of this study and our model demonstrate that allele-specific maintenance of the centromeric 11p15 domain is not limited to the ICR itself, but involves epigenetic elements across the region.

However, one question presented by these interactome findings is how the familial *KCNQ1* 5′ deletion, which lies between but does not encompass Regions1/3 can abrogate CTCF binding and alter the ICR methylation status. Imprinting methylation marks are established during gametogenesis when chromatin is compacted for cell division and is not generally organized by topological domain (3,74). Therefore, it is unlikely that CTCF-mediated loops are responsible for the constitutive failure to establish IC2-ICR methylation in the III-3 *KCNQ1* 5′ deletion patient. Previous studies in mouse and human have demonstrated the importance of the sense and antisense transcription of *KCNQ1* and *KCNQ1OT1*, respectively (20,28–33). Several studies report the silencing ability of *KCNQ1OT1* in a bidirectional manner across the domain (28,31,33,80–83). Yet, one role of antisense transcription at other imprinted loci is promoter competition (84), and the *Kcnq1ot1* transcript does not proceed through, and therefore cause interference with, the *Kcnq1* promoter (35,85). Interestingly, it has been noted that knockdown of the *Kcnq1ot1* transcript post-transcriptionally does not disrupt the imprint (86). A study by Golding *et al.* suggests that paternal transcription initiation or chromatin opening is more important than the antisense transcript itself (86). The results we present in our study support this conclusion in that both BWS LOM and III-3 *KCNQ1* 5′ deletion samples demonstrate biallelic expression of *KCNQ1OT1* although this is not accompanied by an increased transcript abundance in the case of the BWS LOM group (Figure 3C–E).

From the sense perspective, truncation of *Kcnq1* transcription prior to reaching the DMR resulted in near complete LOM and biallelic *Kcnq1ot1* transcription (33). The human deletion reported by Beygo *et al.* (19) also maps upstream of the ICR-DMR, encompasses the *KCNQ1* TSS, and leads to ICR LOM similar to the smaller deletion reported in this study (Figure 2A). They propose abrogated *KCNQ1* transcription during oocyte development leads to the imprint failure (19). Our study supports this conclusion and we further report resultant increased *KCNQ1OT1* expression as a result of the structural alteration (Figure 3A,C), suggesting a *KCNQ1/KCNQ1OT1* antagonistic effect even without promoter competition. Further studies in human and hybrid mouse models are required to better understand the interplay and role of sense versus antisense transcription as a regulator of allele-specific methylation deposition during gametogenesis, as well as in methylation maintenance in somatic tissues.

## DATA AVAILABILITY

Data are accessible in dbGAP (phs002408.v1.p1).

## Supplementary Material

gkab475_Supplemental_Files
