# Supplementary material for: The role of CTCF in the organization of the centromeric 11p15 imprinted domain interactome
Source: Nucleic Acids Res. 2021 Jun 9;49(11):6315–30. doi: 10.1093/nar/gkab475 (PMC8216465; doi:10.1093/nar/gkab475)

Figure S1. Characterization of the 7.6 kB familial deletion. A) Copy number variation (CNV) data from aCGH indicating a loss of 6.8 kB sequence from the 5' end of the KCNQ1 gene within the BWS region on chr11p15. B) Whole Genome Sequencing (WGS) data mapped to hg19 for the proband indicates decreased read depth across the chr11:2466050-2473630 interval visualized in IGV. C) Consistent read depth along this interval is observed in the Control3 fibroblast sample and 50% read depth is observed in the III-3 fibroblast sample, which confirms the location and full length of the heterozygous deletion in the III-3 sample.

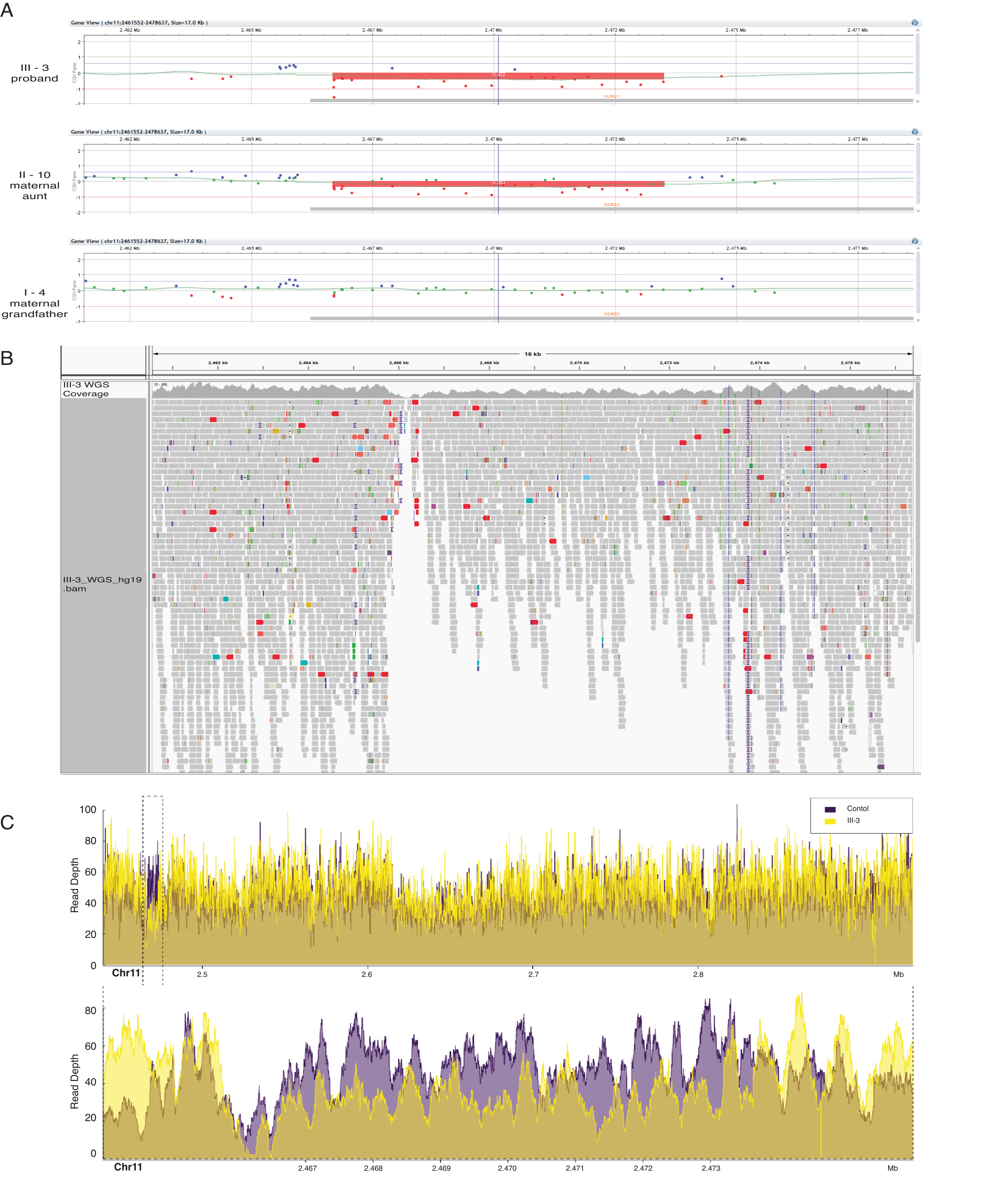

Figure S2. CTCF binding in mouse is conserved with that of human and is biallelic. A) mm9 UCSC Genome Browser view of chr7:150,257,304-150,662,914 including Kcnq1, Kcnq1ot1, and Cdkn1c indicated in purple within the human-orthologous IC2 domain. CTCF binding sites in CH12 cells are indicated in light blue and binding tracks from an allele-specific CTCF ChIP-seq are indicated in gray. B) IGV browser view of variants from allele-specific CTCF ChIP-seq across the region highlighted by light blue in Kcnq1 intron2 in panel A demonstrating biallelic binding through equal capture of single nucleotide variants in CTCF binding.

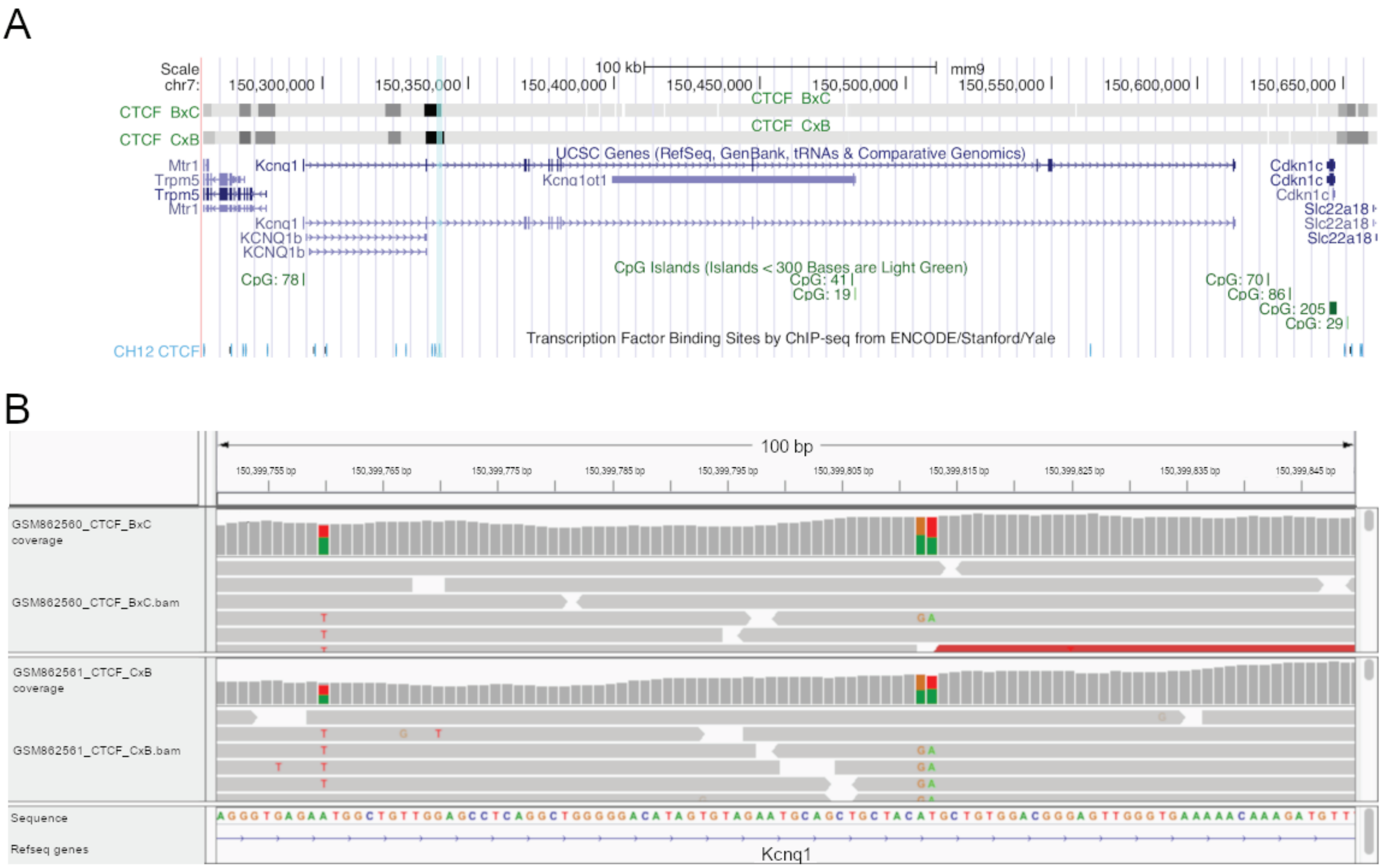

Figure S3. Interaction profiles between IC1 and IC2 probes do not show interaction between ICRs directly. Panels display hg19 chr11:1,982,954-2,936,213. A) HiC interactome from GM12878 cells at 5kB resolution of both 11p15 imprinted domains. DNase Hypersensitivity Sites (DHSs) are displayed. B) UCSC Genome Browser view of the HiC region with genes indicated in purple, CpG islands indicated in green, and CTCF ChIP-seq binding in GM12878 cells indicated in red. Interactions across 11p15.5 imprinted domains do not connect IC1 to IC2 in C) control fibroblasts (N=3), D) BWS LOM fibroblasts (N=3), E) and III-3 KCNQ1 5' deletion fibroblasts.

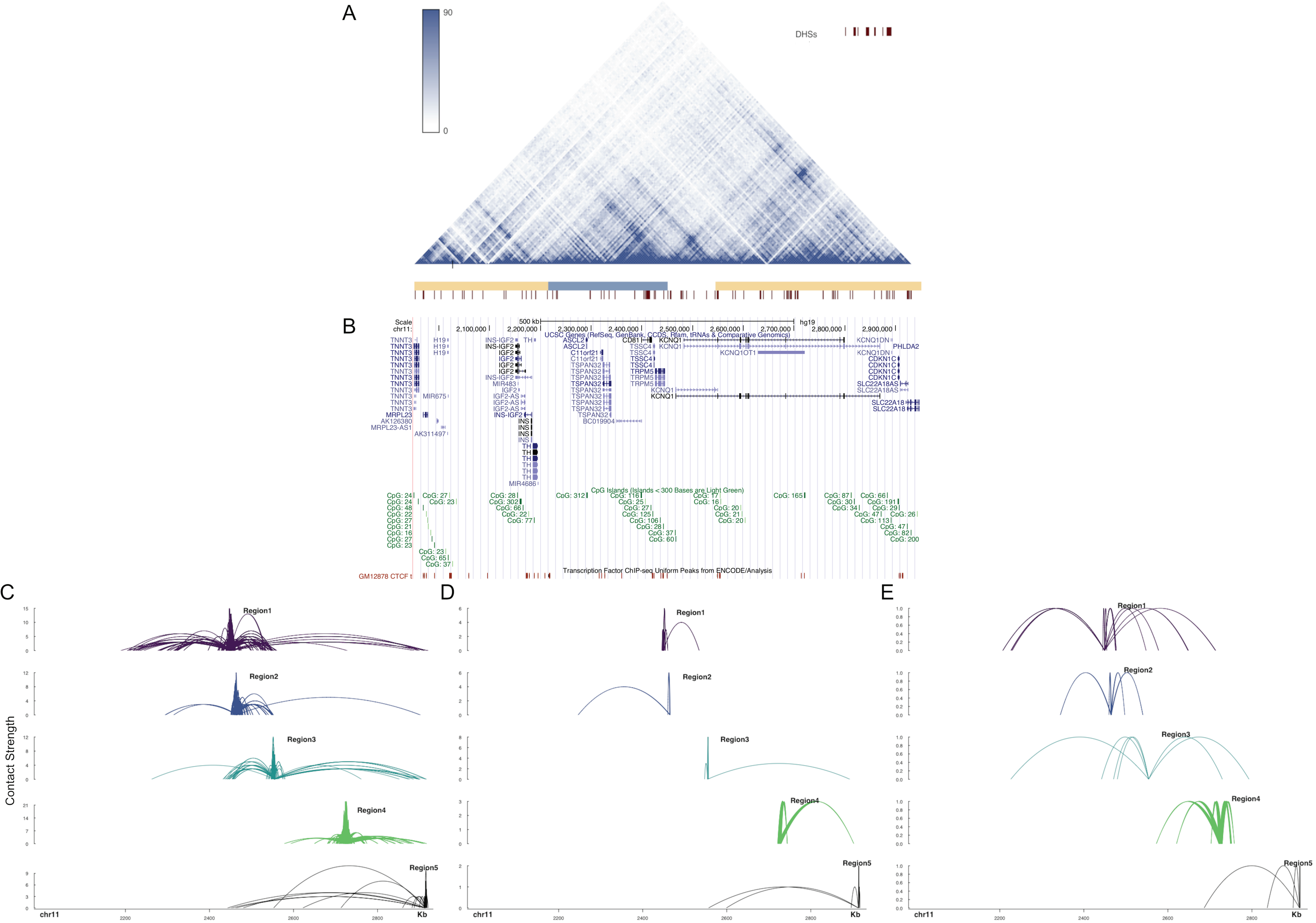

Figure S4. HiC interactome of mouse BWS-orthologous imprinted domains. mm9 UCSC Genome Browser View and the HiC interactome from CH12 cells at 5kB resolution of chr7:149,720,000-150,950,000 contains contact depletion between mouse IC1 and IC2 imprinted domains.

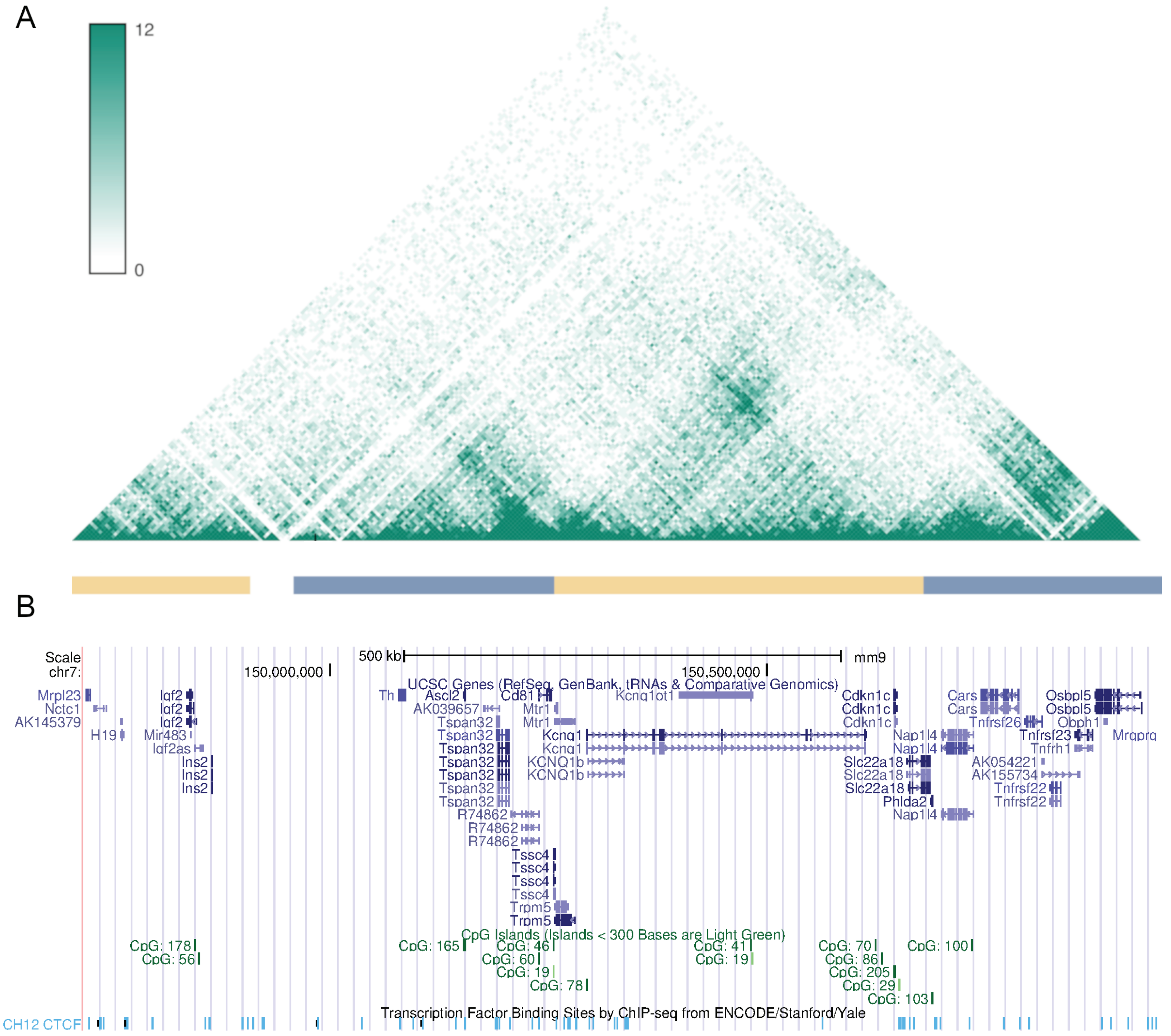

Figure S5. Trans interactions of other IC2 probes across the genome. Common features of control fibroblasts (N=3) are shown in green, common features of BWS LOM fibroblasts (N=3) are shown in yellow, and III-3 KCNQ1 deletion fibroblast profile is shown in blue. Trans interactome of the A) Region1, B) Region2, C) Region3, and D) Region5 anchors are displayed. Interactions across chromosome 11 are not shown.

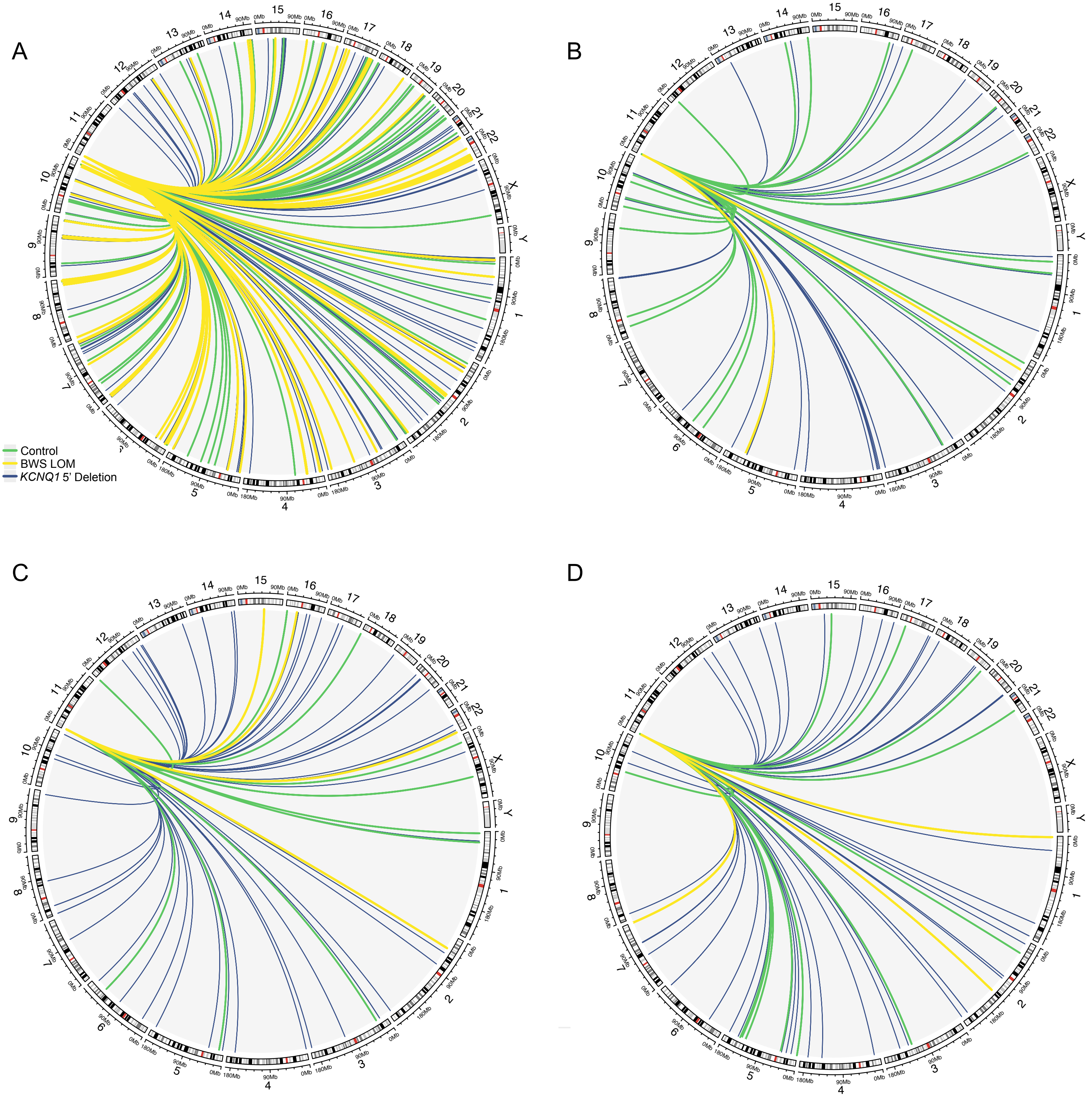

Supplement: gkab475_Supplemental_Files [file gkab475_supplemental_files.zip › SobelNaveh_etal2020-supplementalFigsV2.pdf]
