# Supplementary material for: The role of CTCF in the organization of the centromeric 11p15 imprinted domain interactome
Source: Nucleic Acids Res. 2021 Jun 9;49(11):6315–30. doi: 10.1093/nar/gkab475 (PMC8216465; doi:10.1093/nar/gkab475)
Supplement: gkab475_Supplemental_Files [file gkab475_supplemental_files.zip › TableS1.primersRevised2.docx]

**Supplementary Table 1. Primer and probe sequences used in this study.**

| Name | Sequence (hg19) | Application |
| --- | --- | --- |
| Region1-left | GATCCTGCATAGCTGTTGAGCTACTTAGCCACTTGGGCCTCAGTACCTTGGAGTGTCAAACAGGGACTCTAATAGCCTGGCTTCTGGGAGGTTGCAGGTAACACATGAGGAGAACAGAGCCTGGG | CaptureC |
| Region1-right | GGCAGAGTCTGAGGCAGTGGTGGTAACGGGTGAAGGTGATGGTGGTGGTGGTGATGATGGTGGCAGTAGTGGTGGTGGTGGTGATGATGATATTGATGGTCATGATGGTGGTGGTGACAATGATC | CaptureC |
| Region2-left | GATCGAAACTGCAAATACTTTTCCCATTTGGTCATTTGTCTCCTTTACTTTGCTAATGGTGCTTCTATTTTAATTTTTCTTATTTTATCTGTTGCTTTTGGATTTCAAGTCATAGTTGGAA | CaptureC |
| Region2-right | TACCTGACAGTGGTGGTCCCATCTCACCCTCCCGGTCCAAGGGCCCTGGTGGGAAGCGACCCGAGCGTATTCGCCGCCCGCAGCTCGCGCGCCTGGGCACCCGGGGGCGCTCACAGTGATC | CaptureC |
| Region3-left | GATCAGAGCACAGGCAGAAGTCACCTGACAGCAGGTTCCCGAGGCTTCAGGCCTGGAGGAGTGAGGACGGCTGTTGGGTTCTTGATTCCAGCAGAGGGAGGCTGTGCAGGGTTGCAGGAAG | CaptureC |
| Region3-right | TGCTGAGCGCGGGGAGCCCAAACCACAGCCTTGGGGAACCCAGGAGGGGCCGGGCGGGTGCAGGACCCGCATGAGGGATTCCGAGAGCACGATGTTTAAATGTTGGGGGAAAAAATGGATC | CaptureC |
| Region4-left | GATCACCCGTCCCGCGCCGTCCGCCCAGGTCCGTGCTGACCGTGTTCAAACCCTCCCAGAGAGATGGGGAGGGCCGCGCTGAGGAGAGTCTGGGAGAACCGCACTGAGGAGCCGCCGGGAG | CaptureC |
| Region4-right | GACGCACCGAGGAGGACCGCGCTGAGGGGCGCACCGGGAGAATCGTGCTGAGGAGCCCCGGGGAGGACCACGCTGAGAGGCACCCCGGCAGAATCGCGCTGAGGGGCGCCCTGGCAGGATC | CaptureC |
| Region5-left | GATCTCCCTTCCCACCAAGCCCCCTGACTTCCTGCTGGAGCTCGGGGAGACCCCTGTGACCAGCATCTCCTGCCTACGGGGGGCGCCAGGGCCTCAAAGCCTCTCCAAGCCACTCCCTCCCACCC | CaptureC |
| Region5-right | GGCCAGGGCCTTGCTCTCTGGCCCCGAGTCCCCTCAAGTGTAAGATGGGGGCTCTGCCCAGTGTGGCTGTCCGGGCTGCAGCGGGCAGGAGCGCATTTTTCACAAACCCCAACACCCCTCGGATC | CaptureC |
| ANXA9-Forward | TCTCCTGCATGTGTCTGAGG | ChIP |
| ANXA9-Reverse | CTAGTGGCCCCTTGAGGTC | ChIP |
| CTCF1-Forward | TCCCAAAGAGACTCCAAGGAC | ChIP |
| CTCF1-Reverse | ACCATCACCTTCACCCGTTA | ChIP |
| CTCF3-Forward | CGGCTGTTGGGTTCTTGATT | ChIP |
| CTCF3-Reverse | AGCTCCTGAGTTGCATGTAG | ChIP |
| CTCF5-Forward | GAACGCTGTACACCACTTCC | ChIP |
| CTCF5-Reverse | CAACACAGGCAATGCGGT | ChIP |
| ACTB-Forward | ATCAAGATCATTGCTCCTCCTG | qRT-PCR |
| ACTB-Reverse | AGTCCGCCTAGAAGCATTTG | qRT-PCR |
| RPLP0-Forward | CTCGTGGAAGTGACATCGTC | qRT-PCR |
| RPLP0-Reverse | GTCTGCTCCCACAATGAAAC | qRT-PCR |
| KCNQ1-Forward | CATCTCCTTCTTTGCGCTCC | qRT-PCR |
| KCNQ1-Reverse | GGCCTTCCGGATGTAGATCT | qRT-PCR |
| KCNQ1OT1-Forward | GGGAGCTGTTGTCCCTTACC | qRT-PCR |
| KCNQ1OT1-Reverse | TTCGGAGTGGTAACTGTGCC | qRT-PCR |
| CDKN1C-Forward | GGGCCTCTGATCTCCGATTT | qRT-PCR |
| CDKN1C-Reverse | TTGGGCTCTAAATTGGCTCAC | qRT-PCR |
| KCNQ1OT1- rs231362 -Forward | TCCCCAAGTCACAACCTCAA | Sanger |
| KCNQ1OT1- rs231362 -Reverse | CTGTGTGTCTCAGCCAACAG | Sanger |
| CTCF3- r s67439072-Forward | TGTAACACTACCCACCCCAC | Sanger |
| CTCF3- r s67439072-Reverse | TGACAGCTGGACCCTCAAAT | Sanger |
